# Bioconjugation of Vegetable Oils with UV Absorbers: New Approach in Skin Photoprotection

**DOI:** 10.3390/molecules28227550

**Published:** 2023-11-12

**Authors:** Andrei Iulian Slabu, Laura Miu, Emilian Ghibu, Cristina Elena Stavarache, Raluca Stan, Florina Teodorescu

**Affiliations:** 1 “C. D. Nenitzescu” Institute of Organic and Supramolecular Chemistry of the Romanian Academy, 202 B Spl. Independenței, S6, 060023 Bucharest, Romania; andrei.slabu@ccocdn.ro (A.I.S.); laura.miu@ccocdn.ro (L.M.); emilian.ghibu@gmail.com (E.G.); cristina.stavarache@upb.ro (C.E.S.); 2 Faculty of Chemical Engineering and Biotechnologies, National University of Science and Technology Politehnica of Bucharest, 1-7 Gh. Polizu Street, 011061 Bucharest, Romania; raluca.stan@upb.ro; 3 Advanced Polymer Materials Group, National University of Science and Technology Politehnica of Bucharest, 1-7 Gh. Polizu Street, 011061 Bucharest, Romania

**Keywords:** bioconjugation, sea buckthorn oil, *p*-methoxycinnamic acid, oleogel, sun protection factor

## Abstract

We reported the tunable synthesis of new vegetable oil–UV filter bioconjugates using sea buckthorn oil (SBO) and *p*-methoxycinnamic acid (*p*-MCA) as an alternative to the common UV filter, ethylhexyl-*p*-methoxycinnamate (octinoxate). The synthetic strategy is based on the sustainable ring-opening reaction of epoxidized SBO with *p*-MCA in heterogenous catalysis in eco-friendly solvents. The amount of UV-absorptive moieties grafted on the triglyceride backbone is controlled by different epoxidation degrees as determined by NMR spectroscopy. The performance of the new UV-absorber bioconjugates was assessed by in vitro sun protection factor (SPF) measurements after inclusion in SBO-ethylcellulose (EC) oleogels and comparison with the SPF value of the SBO-EC-octinoxate oleogel with equivalent *p*-MCA acid moieties (10% wt/wt). The concentration obtained for the SBO-EC oleogel formulated with the bioconjugate with the lowest degree of functionalization, namely 55%, represents 45% of the SPF determined for the SBO-EC-octinoxate oleogel, regardless of the concentration of measured solutions. The new concept of vegetable oil–UV-absorber bioconjugates has potential UV-B photoprotective properties when included in oleogel formulations and deserves further investigation of their properties and stability including association with UV-A absorbers, respectively.

## 1. Introduction

Climate change has made sun protection factor (SPF) products a need in everyone’s life since they shield the skin from potential inflammations, sunburns, early aging, and skin cancer. Cinnamic acid derivatives are among the most often used excellent solar filters because they effectively absorb UV radiation, particularly in the UV-B range. Ethylhexyl methoxycinnamate (octinoxate), the most popular solar filter, is used in sunscreens to absorb UV-B rays. To protect the skin from UV-A rays, this compound is typically coupled with titanium oxide, oxybenzone, or avobenzone. However, research on octinoxate remains ongoing because of concerns about its potential for endocrine disruption [1,2], but mostly due to low UV resistance leading to photoinduced degradation both in solution and especially in its aggregated form determined by the formation of films on skin surface [3]. The resultant photodegradation products, for example, *p*-methoxybenzaldehyde and two stable cyclodimers, have a decreased ability to absorb UV-B radiation and present cellular toxicity [4]. The UV resistance of octinoxate may be enhanced by encapsulation in lipid or mesoporous silica carriers, synthetic polymers, or ethylcellulose nanoparticles [5].

On the other hand, also from the cinnamates class, *p*-methoxycinnamic (*p*-MCA) acid attracted attention because it has significant UV-B absorbance, making it an excellent candidate for solar filters in cosmetic items. It is also known for its biological properties and for its applications in medicine, such as being an antimicrobial, antidiabetic, or neuroprotective agent [6]. This compound can be extracted from cinnamon, along with cinnamic acid, being then selectively separated by permeation through liquid membranes, but it can also be chemically synthesized. *p*-MCA is also widespread in different varieties of coffee and cereal plants (thus being a compound ingested through food) and in different leaves and roots of plants, like pineapples and banana, making it a biologically active ingredient with the ability to be metabolized by natural microorganisms. Consequently, *p*-MCA is a valuable, attractive compound for diverse industrial applications owing to its nontoxic, useful biological properties [7].

Bioconjugation of lipids with biologically active molecules, for example, phenolic antioxidants, yields potentially new therapeutic molecules with increased bioaccessibility and positive pharmacological evaluation on oxidative stress, skin regeneration, and anti-inflammatory properties [8].

In the context of transitioning from traditional to green chemistry, there is increased interest in switching from petroleum-based products to natural ones, bringing in foreground vegetable oils (VOs), which are not only a non-polluting renewable resource, but one of the most demanded bioresources [9]. Being a valuable source of triglycerides whose composition in fatty acids (FA), especially unsaturated FA, varies based on the environment, region, and growth period of the plant [10], VOs have numerous applications, being increasingly used in industrial applications like cosmetics, soaps, plasticizer, paints, biofuels, etc. [11]. Due to their specific fatty acid profile and the content of their bioactive molecules, vegetable oils are considered as promising starting materials for biomedical applications such as membranes, nanocarriers, fibrous scaffolds, oleogels, and bigels [12].

Sea buckthorn (*Hippophae rhamnoides* L. or *Elaeagnus rhamnoides* (L.) A. Nelson) oil isolated from berries’ pulp or seeds is a valuable resource due to its unique fatty acid composition rich in PUFAs (polyunsaturated fatty acids) and additional content of fat-soluble vitamins, carotenoids, and sterols [13]. It is well known that sea buckthorn oil (SBO) has good vitalizing characteristics that boost appetite, which makes it suitable in food applications [14,15]. On the other hand, it has multiple applications; for instance, it is used in cosmetic products as a good emollient, anti-ageing [16] ingredient. Moreover, in vitro studies proved that SBO is a promising natural substance in skin photoprotection due to prevention of UV impairment in redox systems and lipid metabolism disorders in skin fibroblasts and keratinocytes [17]. SBO was successfully employed in the formulation of nanostructured lipid carriers with cell regeneration effects, promising antioxidant and anti-inflammatory activity [18], and improved skin hydration effect [19] and, recently, was formulated as edible oleogels using carnauba wax or beeswax [20].

We have recently reported sustainable functionalization of vegetable oils with organic acids (including cinnamic acid) via epoxy ring-opening reactions using recoverable heterogeneous catalysts (layered double hydroxides—LDH) and environmentally friendly bio-solvents [21]. This concept is further extended and reported in this paper for the tunable synthesis of *p*-methoxycinnamic acid (*p*-MCA)–vegetable oil (SBO) bioconjugates to obtain new compounds with UV photoprotective effects having different *p*-MCA functionalization degrees controlled by the initial epoxidation of the starting material. The designed bioconjugates present the same conjugated motif as octinoxate to ensure UV-B absorption but, due to their structure, might be a more photostable and skin-protective alternative. The selected synthetic approach of attachment of UV-absorbing *p*-MCA moieties on the epoxidized skeleton of triglycerides from SBO was chosen according to the hypothesis that the tridimensional structure of the triglyceride backbone will prevent association of *p*-MCA into aggregates that, according to the literature data, favor photodegradation of parent compound octinoxate [3]. The alternative of bioconjugation of *p*-MCA with a single chain of a fatty acid, as reported for other bioactive molecules [8], favors aggregation of the new UV filter through additional van der Waals non-covalent interactions between hydrophobic aliphatic chains, which might enhance the photodegradation of the bioconjugate and, as a consequence, was not taken into consideration. Moreover, the significant molecular mass of the designed bioconjugates and their increased hydrophobicity will prevent percutaneous absorption of the UV absorber and the subsequent effects on humans. According to the literature data, detectable levels of octinoxate have been found in blood, urine, and breast milk due to systemic absorption, although the determined levels are much lower than those in in vitro experiments [22].

The new UV-absorber bioconjugates were tested by including them in SBO-ethylcellulose (EC) oleogels and their performance was assessed by SPF measurements. This type of formulation was chosen due to the oleogels’ ability to alleviate cracked skin, provide protection against UV rays and cold temperatures, and its water and perspiration resistance important for the development of a sunscreen formulation. Ethylcellulose was selected as the organogelator due to its extensive use in obtaining food-grade oleogels [23] and for its reported protective effect on octinoxate [24] and vegetable oil [25].

Based on all the available information, we intended to obtain *p*-MCA-SBO bioconjugates with similar tridimensional geometry to the SBO used in oleogel formulations, thus ensuring a good compatibility and, as a consequence, a good distribution of the UV-absorber moieties within the oleogel mass.

## 2. Results

**Figure 1:**
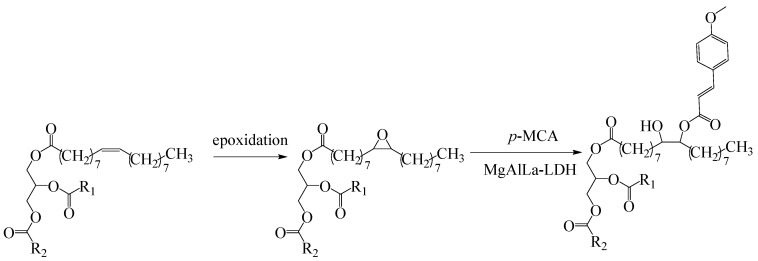
The functionalization stages of sea buckthorn oil.

The synthetic approach to obtain the new bioconjugates is summarized in Figure 1: firstly, SBO was epoxidized using the previously reported method [21] followed by ring-opening reactions in the presence of MgAlLa-LDH catalyst.

In the next section, each epoxidized sea buckthorn oil (ESBO) product will be annotated as follows: ^x^A, where x represents the degree of epoxidation. The annotation for functionalized products of ESBO—the new synthesized bioconjugates with *p*-methoxycinnamic acid (named *p*-MCA-SBO)—will be ^x^B, having an x epoxidation degree and a 100% functionalization with *p*-MCA degree. For all the oleogels, the annotation will be ^x^B_y_, where ^x^B is the functionalized product added (previously detailed annotation) and y is the amount of *p*-MCA in the final product (as exemplified in Table 1).

### 2.1. Epoxidation Reactions

In order to obtain relevant information concerning the epoxidation reaction, samples were taken and characterized by ^1^H RMN, showing partial degrees of epoxidation at different times and quasi-total epoxidation after approximatively 5 h. As it can be seen in Table 2, the results are reproducible and present a standard deviation below 1, which shows a low variation. The graph also indicates that a desired epoxidation degree may be obtained based on reaction time.

### 2.2. Ring-Opening Reactions

The ring-opening reactions of the ESBO occur differently depending on the epoxidation degree (as mentioned in Section 4.5), and as the epoxidation degree gets smaller, it gets more difficult to open the epoxy ring with *p*-MCA.

Therefore, even though the lower the degree of epoxidation, the more difficult it is to open the epoxy rings, the total epoxy ring opening is confirmed by ^1^H NMR analysis (Figure S1). The ^1^H NMR spectra show the disappearance of the signals corresponding to the protons involved in the epoxy ring from 2.89 to 3.11 ppm, and the appearance of signals corresponding to protons from the aromatic ring (6.89 and 7.65–7.67 ppm) and from the methoxy group (3.84 ppm) of the opening reagent. Nevertheless, all epoxidation products were 100% functionalized with *p*-MCA, and the products were used in the following experiments as potential UV absorbers added in oleogels.

### 2.3. Oleogel Preparation

Cinnamic acid, based on previous literature studies, has great potential as an organogelator [26]. As *p*-MCA is a cinnamic acid derivative, to make the oleogel formulation more compact, in preliminary gelification essays, it was added to the oleogel as such, or as an oil functionalized with *p*-MCA, but no gelation was observed. For the purpose of gelation, ethylcellulose, the well-known polymeric organogelator for being a good gelling agent for VOs in many applications, commercially available, , was used. In the cosmetic field, it can also be used as a UV filter carrier in bio-based functionalized nanoparticles [27]. Also, according to the literature [28], being an efficient gelling agent, the content of EC varies between 0.1 and 10% of the final product. Gelling tests were performed in order to use as minimal EC as possible, which resulted in a minimum of 3 wt%. EC necessary for gelation of the SBO. This percentage also allows the dissolution of SBO-EC-based formulations in pure ethanol [29].

### 2.4. The SPF Values Correlation

Firstly, the SPF values of ^100^B_y_, ^80^B_y_, and ^55^B_y_ were determined at the concentration of 0.2 mg/mL ethanol solution. After determining that ^55^B_10_ has the highest SPF value, a positive and a negative control study was performed by analyzing SBO and SBO-EC-octinoxate oleogels, respectively, at the same concentration. While the SPF value of SBO was only 0.6, the presence of the *p*-MCA moieties in the structure of the ^55^B_10_ increased the SPF value up to 7.2; this value represented 45% of the SPF value of SBO-EC-octinoxate oleogel (15.8), the new UV-absorber bioconjugate providing promising UV filter properties.

**Figure 2:**
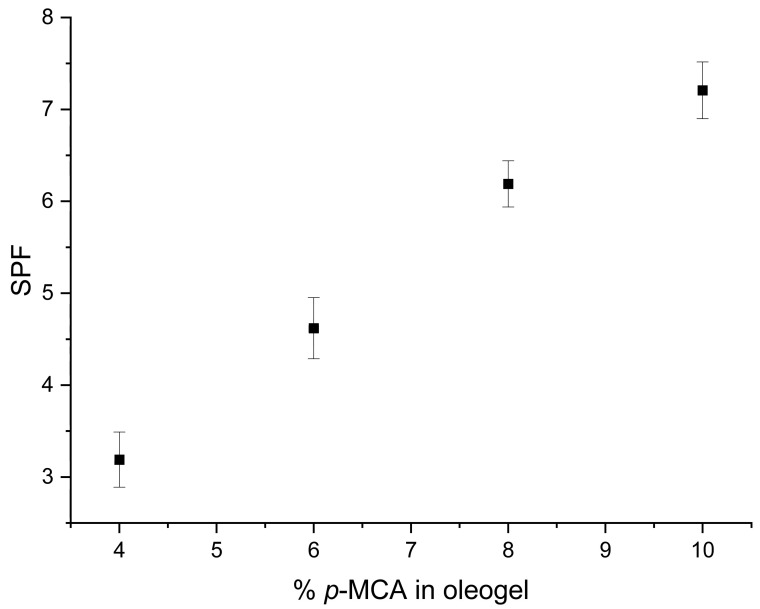
The increase in ^55^By’s SPFs at 0.2 mg/mL concentration (standard deviation below 1).

The incorporation of different amounts of UV-absorber bioconjugate into oleogels led to an expected increase in SPF values, which is correlated with the increase in the amount of *p*-MCA (y) in the oleogel (Figure 2) within the limits accepted by the European Commission for octinoxate, as previously stated [30]. For instance, the SPF values for ^55^B_y_ at 0.2 mg/mL oleogel solution concentration (Figure 2) are directly proportional with the *p*-MCA amounts in oleogel (standard deviation below 1, as can be seen in Table S1), as expected.

**Figure 3:**
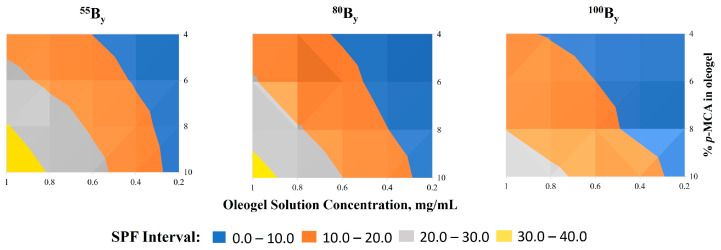
The correlations of the SPF values of oleogels according to the UV absorbers and the modified parameters.

The appropriate selection of different parameters, such as the epoxidation degree of the UV absorbers, the amount of *p*-MCA from the oleogels, the oleogel solution concentration, and the adequate correlation between them, lead to the possibility of obtaining a bioconjugate product with an SPF in the desired value range, as can be seen in Figure 3.

## 3. Discussion

### 3.1. Epoxidation Reaction

From a qualitative point of view, three epoxidation products, with 55%, 80%, and 100% epoxidation of the unsaturated bonds, were analyzed by NMR.

**Figure 4:**
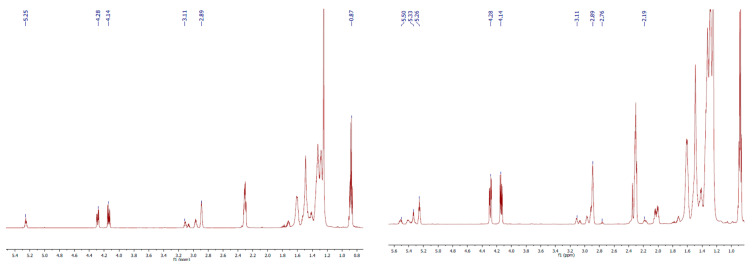
The ^1^H NMR spectrum of ^100^A (**left**) and ^80^A (**right**).

From the ^1^H NMR spectrum of the ^100^A, the disappearance of all signals corresponding to the protons of double bonds, assigned at 5.34 ppm, and the appearance of the signals from 2.89 to 3.11 ppm can be noticed. These signals are correlated to the protons in the epoxy ring, proving that all the double bonds have been completely epoxidized (Figure 4). On the other hand, the ^1^H NMR spectrum of the ^80^A exhibits small signals at 5.34–5.5 ppm, and simultaneously, the total disappearance of the signal corresponding to the protons between two double bonds, assigned at 2.76 ppm (the signal corresponding to the linoleic chains), indicating that all the triglyceride branches corresponding to the polyunsaturated acids have been fully epoxidized (Figure 4).

**Figure 5:**
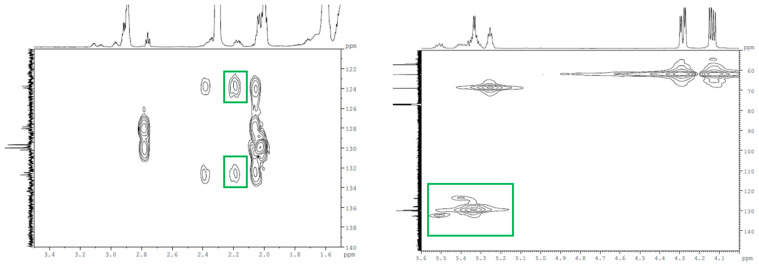
The 2D HMBC (**left**) and the 2D HMQC (**right**) spectrum of ^55^A.

From the 2D COSY spectrum of the ^55^A, the couplings of the resonance signal from 2.18 ppm (attributed to –CH_2_, the protons between one epoxy ring and one double bond) with the signal from 2.92 ppm (corresponding to the epoxy ring—Figure S1) and the signals from 5.34 to 5.5 ppm (attributed to the double-bond protons –HC=CH–—Figure S1) can be observed. Also, the carbons with the double bond are assigned in 2D HMQC at 123.7 and 132.5 ppm (Figure 5). The 2D HMBC spectrum shows the cross-peaks from the correlation of the double-bond carbons with the resonance signals of the protons at 2.18 ppm (Figure 5). From all this information, it can be assessed that non-epoxidized double bonds (–HC=CH–) are near the glycerol backbone in the partially epoxidized samples. This hypothesis is supported by previously reported experimental data on epoxidized vegetable oils [31], which showed that the marginal double bonds of triglycerides are the first to be epoxidized. Examining the NMR spectra of ESBO, no degradation products of the SBO, for example aldehydes or acids, were identified.

### 3.2. Ring-Opening Reaction

Previously reported experimental data on epoxidized vegetable oils [31] showed that, in the case of an oil with a lower epoxidation degree, mostly on the marginal double bonds, the functionalization should not be impeded; this cannot be observed in the ESBO experiments. An explanation of this behavior might be provided by the structure of *p*-MCA and its tendency to associate through π-π stacking interactions, as can be seen from its RAMAN spectrum (Figure S2), where a single characteristic band for C_ar_-H in-plane bending vibrations found at 1171.8 cm^−1^, due to restricted mobility, resulting from aggregation is observed. On the contrary, in the RAMAN spectrum of bioconjugate ^100^B with the maximum degree of functionalization (Figure S3), part of the aromatic rings of *p*-MCA are no longer in a favorable position for association through π-π stacking [32], as revealed by the presence of the characteristic absorption of C_ar_-H in-plane bending vibrations as a doublet at 1249 and 1243 cm^−1^. We presumed that in the case of bioconjugate ^55^B synthesis during functionalization by ring-opening reaction, the first *p*-MCA moieties are attached to the more accessible marginal epoxy rings and the resulting structure still presents a good configuration for association via aromatic rings, thus compacting the molecules and acting as the steric hindrance factor for further functionalization.

All this information can also confirm that, in the gelling tests (presented in Section 2.3), the self-assembly of the cinnamic moieties from *p*-MCA and the functionalized products has not occurred as in the case of cinnamic acid [26], and that is why *p*-MCA did not function as a gelling agent. Moreover, according to the literature [33], triglycerides with a high content of oleic residues, like triolein, also present in raw SBO, tend to self-associate, forming a compact structure and providing supplementary steric hindrance for functionalization. The self-assembly of the cinnamic moieties and the position of the non-epoxidized double bonds (near the glycerol backbone, equivalent to the oleic acid) could explain why the ring-opening reactions for partially epoxidized SBO are so difficult.

### 3.3. The UV-Absorber Bioconjugate Photoprotective Activity

The obtaining of a predetermined sun protection factor depends on three parameters: (i) the desired amount of *p*-MCA from the final product (within the limits accepted by the European Commission, as previously stated [30]), (ii) the epoxidation degree, and (iii) variations in oleogel solution concentration. After the examination of SPF values, from the first parameter point of view, the sun protection factor shows a linear increase (as can be seen in Figure 2, Section 2.4). The second parameter is very important because the SPF increases as the epoxidation degree decreases. So, for instance, in ^55^A, the double bond furthest from the ester group was first epoxidized, as explained in Section 3.1. This implies that the *p*-MCA attached in the next step, in the ring-opening reaction, was accessible, being far from the glycerol backbone. This could lead to a high absorption rate when the oleogel that contains ^55^B as a UV absorber is analyzed under UV.

Based on the third parameter, for each oleogel obtained, the SPF was measured at 5 different concentrations. The UV analysis shows increasing SPF values from the diluted solution to the concentrated one for all the oleogels. For example, ^80^B_4_ analyzed at a concentration of 0.2 mg/mL presents an SPF value of 2.9, while at a concentration of 1 mg/mL, it has an SPF value of 14.8 (Figure 3).

There is also a difference in SPF values between oleogels that have the same amount of *p*-MCA and were analyzed at the same concentration, but which contain *p*-MCA-SBO with different degrees of epoxidation. For example, for the oleogels analyzed at concentration of 1 mg/mL, the SPF value was 16.6 for the ^55^B_4_, while for ^100^B_4_, it was 11.2. In the same correlation, the SPF value was 35.8 for ^55^B_10_, while for ^100^B_10_, it was 26. These values demonstrate that the lower the degree of epoxidation of the bioconjugate is, the higher the SPF value of that oleogel will be for the same amount of *p*-methoxycinnamic acid found in the final oleogel.

## 4. Materials and Methods

### 4.1. Materials

Malonic acid, anisic aldehyde, pyridine, piperidine, hydrochloric acid, technical limonene (dipentene), ethylcellulose (48%, 22 cps), glacial acetic acid, sulfuric acid (95–97 vol.%), and technical grade toluene were purchased from Sigma-Aldrich (Saint Louis, MO, USA). The sea buckthorn oil, obtained by cold-pressing process, was provided from a local company (56% oleic acid). Hydrogen peroxide (30 vol.%) was purchased from Atochim SRL (Bucharest, Romania). *p*-methoxycinnamic acid was synthesized in the laboratory. MgAlLa LDH was provided by the Department of Organic Chemistry, Biochemistry and Catalysis, University of Bucharest.

### 4.2. Methods

#### 4.2.1. Nuclear Magnetic Resonance (NMR) Spectrometry

^1^H NMR was used for studying the structures of SBO, ESBO, and *p*-MCA-SBO. The samples were dissolved in 0.5 mL CDCl_3_, and the spectra were recorded using a Bruker Advance III (Billerica, MA, USA) 600 MHz spectrometer with a resonance frequency of 600.12 MHz for the ^1^H nucleus, equipped with an indirect detection for nuclei probe head (BBI) and field gradients on the Z axis. The chemical shifts were measured in ppm, the signals were calibrated using the CDCl_3_ signal (7.26 ppm), and tetramethylsilane (TMS) was used as the internal standard.

#### 4.2.2. RAMAN Spectrometry

RAMAN spectrometry was used to analyze the structures of SBO, ESBO, the ring-opening reagent, *p*-MCA, and *p*-MCA-SBO. A HR Evolution HORIBA (Palaiseau, France) spectrometer equipped with a 514 nm laser was used. The acquisition time was 2 s, accumulation was 20, hole diameter was 100 micro, objective was 50×, grating was 600 gr/mm, and the ND filter was 100%.

#### 4.2.3. UV-Vis Spectrometry

UV-Vis data were acquired on a Varian Cary 100 Bio UV-Vis (Varian Inc., Raleigh, NC, USA) spectrophotometer equipped with a Cary temperature and stir control. Samples were examined in quartz cuvettes with a pathlength of 1.0 cm in the wavelength range of 200–800 nm. This method was used to determine the absorbance of each oleogel and the values were further used for SPF calculation.

#### 4.2.4. Melting Point Measurements

Melting points were determined by thermal analysis using a “Polytherm A” microscope (Boëtius hot plate microscope) connected to a heater with temperature control from Wagner & Munz, Munich, Germany. This analysis was used for the identification and purity testing of *p*-MCA and the melting point obtained was uncorrected.

#### 4.2.5. Graphical Representations

For graphical representations, OriginPro 2022 (64-bit) SR1, version 9.9.0.225, and ChemDraw Professional, version 20.1.1.125, software were used.

### 4.3. Epoxidation of SBO

The epoxidation reaction of double bonds was performed using a previously described method [29] in the following conditions: double bonds/glacial acetic acid/H_2_O_2_ in a molar ratio of 1:2:10, acid catalysis, and toluene as a solvent. Sea buckthorn oil was characterized through ^1^H NMR to ascertain its fatty acid composition, according to the literature [34]. Using this technique, the average unsaturation of the SBO was found to be 2.7 double bonds per triglyceride molecule. The typical reaction is carried out in a three-neck flask equipped with a condenser, thermometer, and a drip funnel for hydrogen peroxide with a magnetic stirrer. For this reaction, 10 mL (10 mmol) of SBO, 3 mL (53 mmol) of glacial acetic acid, and 0.45 mL of sulfuric acid 50% (vol.) were mixed and dissolved in toluene. Then, 26.9 mL (264 mmol) of hydrogen peroxide was added dropwise under continuous stirring at 25 °C. After the reactant has been added, the temperature is increased to 60 °C and the reaction was maintained under these conditions for 22 h, according to the literature [35]. In order to see if, in the case of the sea buckthorn oil, the epoxidation could occur more quickly, the reaction was performed for 24 h and samples were taken at well-established times. For every reaction mixture, the aqueous phase was separated from the organic phase and washed with distilled water several times followed by rinsing with saturated sodium bicarbonate solution. Following that, the organic solvent was evaporated under vacuum and the obtained product was identified and characterized by ^1^H NMR, resulting in ESBO with different degrees of epoxidation.

### 4.4. p-Methoxycinnamic Acid Synthesis

For synthesis of the *p*-methoxycinnamic acid, the reaction was carried out according to the literature [36]. For this purpose, 120 mL (1500 mmol) of pyridine, 12 mL (121 mmol) of piperidine, and 24.5 mL (200 mmol) of *p*-methoxybenzaldehyde were mixed. In this solution, 25 g (240 mmol) of malonic acid was added while continuously stirring, and after that, the mixture was allowed to react at reflux for 4 h. After the reaction mixture had cooled to room temperature, it was added to 160 mL of HCl 10 M aqueous solution on an ice bath. The resulting white precipitate was collected by filtration and washed with water several times, leading to white powdered *p*-MCA with an 89% recovery yield after drying. The purity of *p*-MCA was confirmed by ^1^H NMR and by performing the melting point test, which was 171.7 °C (according to the literature [37], the melting point of *p*-MCA is 171.1–173.2 °C). In the ^1^H NMR spectrum of *p*-MCA, protons were detected in the 3–8 ppm region, with the signals from the aromatic ring at 6.8–7.6 ppm, the olefinic protons at 6.4 ppm and 7.5 ppm, and the methoxy group protons at 3.8 ppm, the latter being the signal of interest in the functionalized product’s spectrum.

### 4.5. ESBO Ring-Opening Reaction

The ring-opening reaction of ESBO was performed according to previous work [21] in heterogenous catalysis using MgAlLa layered double hydroxide (MgAlLa LDH) as a catalyst and *p*-MCA as an opening reagent. In order to obtain *p*-MCA-SBO, 0.925 g (1 mmol) of ESBO, 0.565 g (1.2 mmol) of *p*-MCA, and 0.149 g of MgAlLa LDH (10 wt%) were mixed and dissolved in a green solvent, 10 mL of limonene. Under continuous stirring, the reaction mixture was heated at 170 °C for 48 h. The reaction mixture was then processed by separating the solid catalyst by vacuum filtration, then the filtrate was rinsed with distilled water. After the liquid–liquid separation, the organic solvent was evaporated, and then ^1^H NMR was used to identify and characterize the final *p*-MCA-SBO bioconjugate. The 100% ring opening of partial ESBO has occurred in harsher reaction conditions, such as up to 120 h reaction time and a molar ratio of epoxy rings/*p*-MCA of 1:2; these are the only parameters that can be changed since the raising of temperature above 170 °C is limited by the solvent and the degradation of the oil.

### 4.6. SBO-Based Oleogel Synthesis

For obtaining biobased oleogels, different quantities of reactants were added, in accordance with EU regulations [30] which specify a maximum amount of 10% wt. of cinnamates used as UV absorbers in the final product. Thus, the amount of UV absorber added to the oleogel was calculated to be 4%, 6%, 8%, and 10% *p*-MCA, the UV filter’s active compound, respectively. So, for 1 g of oleogel in a 2.5 mL round-bottom flask equipped with a tight stopper and a magnetic stirrer, 0.03 g (3 wt%) of EC, between 0.12 and 0.44 g (12–44 wt%) of *p*-MCA-SBO bioconjugate, and the difference up to 1 g (53–85 wt%) of the SBO were added. Then, the mixture was purged with argon, heated in an oil bath, and kept under stirring until it reached 145 °C. When the mixture reached the desired temperature, the stirring was stopped, and the mixture was maintained at this temperature for 30 min. The next stage involved cooling the mixture to 120–125 °C and pouring it into a transparent glass container in order to see when, after complete cooling, the oleogel solidifies, forming a translucent orange product [28].

### 4.7. Oleogel’s Analysis and SPF Calculation

The UV spectroscopy was used by measuring the absorbance of a diluted solution of oleogel in ethanol [38], with the solutions being analyzed in triplicate. The oleogel solution concentration varies between 0.2 and 1 mg/mL, correlated to the equivalent concentration of the application density on the skin (x mg/cm^2^) [39]. The sun protection factor was then calculated using the Mansour equation for the UV-B range, 290–320 nm, according to Equation (1)

(1) $$SPF=CF\cdot \sum_{\lambda =290}^{320}EE(\lambda )\cdot I\left(\lambda \right)\cdot Abs(\lambda )$$

where *EE* is the erythemal effect spectrum, *I* is the solar intensity spectrum, *Abs* is the absorbance of sunscreen product, and *CF* is the correction factor (having the value 10) [38].

## 5. Conclusions

In summary, this work showed that sea buckthorn oil may be used to prepare UV-absorber bioconjugates via full or partial epoxidation, with the furthest double bonds from the glycerol backbone of triglycerides being the ones that are first epoxidized, as proved by NMR spectra. The subsequent functionalization with *p*-methoxycinnamic acid by total ring-opening reaction in heterogenous catalysis was influenced by the degree of epoxidation and the π-π stacking interactions of the aromatic moieties observed in the RAMAN spectra. The use of the new UV absorber–SBO bioconjugates as octinoxate replacements in oleogel formulations allows final products with tunable SPF value depending not only on the bioconjugates concentration, but also on their functionalization degree.

The presence of a UV-absorber moiety bioconjugated on the sea buckthorn oil backbone may improve the performance of an oleogel UV-absorber formulation by increasing hydrophobicity and decreasing percutaneous permeation, keeping the protection system on the skin surface but reducing its toxicity and potential harmful side effects. The results showed that the less the oil is epoxidized and fully functionalized with *p*-MCA afterwards, the higher the sun protection factor will be, and thus the oleogel is more capable of protecting against UV-B rays. However, the designed SBO oleogels require future studies to confirm the potential photoprotective effect. The extension of the study with the association of the new bioconjugates with UV-A absorbers should be considered.

## Figures and Tables

**Table 1:** Exemplification of the annotations made.

Test Annotation	Epoxidation Degree, %	% *p*-MCA in Oleogel
^55^A	55	-
^55^B	55	-
^100^B_4_	100	4
^80^B_6_	80	6
^55^B_10_	55	10

**Table 2:** The results of the triplicate experiment of SBO epoxidation.

	The Degree of Epoxidation			
t, h	Reaction 1	Reaction 2	Reaction 3	Distribution
0.5	2.60	3.87	4.01	3.31 ± 0.71
1	33.10	33.39	31.98	32.69 ± 0.71
2	63.80	64.39	64.10	64.10 ± 0.30
3	83.90	83.39	84.20	83.80 ± 0.40
4	93.80	95.39	95.02	94.74 ± 0.79
5	97.80	98.52	97.99	98.16 ± 0.36
6	97.90	98.76	98.10	98.33 ± 0.43
7	98.15	99.00	98.89	98.58 ± 0.42
8	98.40	98.00	99.20	98.60 ± 0.60
24	97.70	98.00	99.10	98.40 ± 0.70

## Data Availability

Data are contained within the article and supplementary materials.

## Supplementary Materials

The following supporting information can be downloaded at: https://www.mdpi.com/article/10.3390/molecules28227550/s1, Figure S1. The ^1^H NMR spectrum of ^100^B; Figure S2. The 2D COSY spectrum of ^55^A; Figure S3: The RAMAN spectrum of *p*-MCA.; Figure S4. The RAMAN spectrum of ^100^B; Table S1. The SPF values for ^55^B_y_, ^80^B_y_, and ^100^B_y_ at different concentrations of *p*-MCA in oleogel.
