# Supplementary material for: Bioconjugation of Vegetable Oils with UV Absorbers: New Approach in Skin Photoprotection
Source: Molecules. 2023 Nov 12;28(22):7550. doi: 10.3390/molecules28227550 (PMC10674893; doi:10.3390/molecules28227550)
Supplement: Supplementary file 1 [file molecules-28-07550-s001.zip › molecules-2681383-supplementary.pdf]

# Supplementary materials of Bioconjugation of vegetable oils with UV absorbers: new approach in skin photoprotection

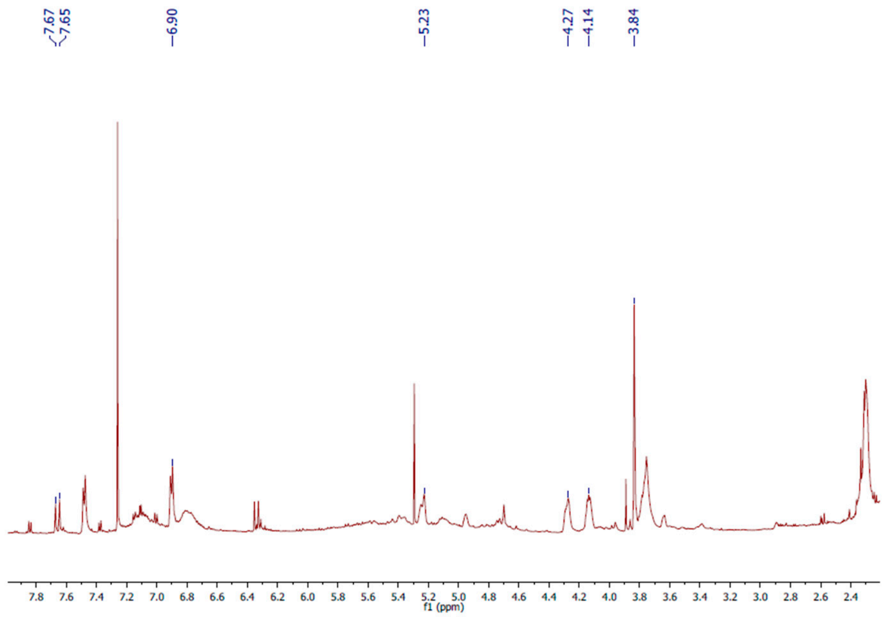


**Figure S1.** The $^{1}H$ NMR spectrum of $^{100}B$

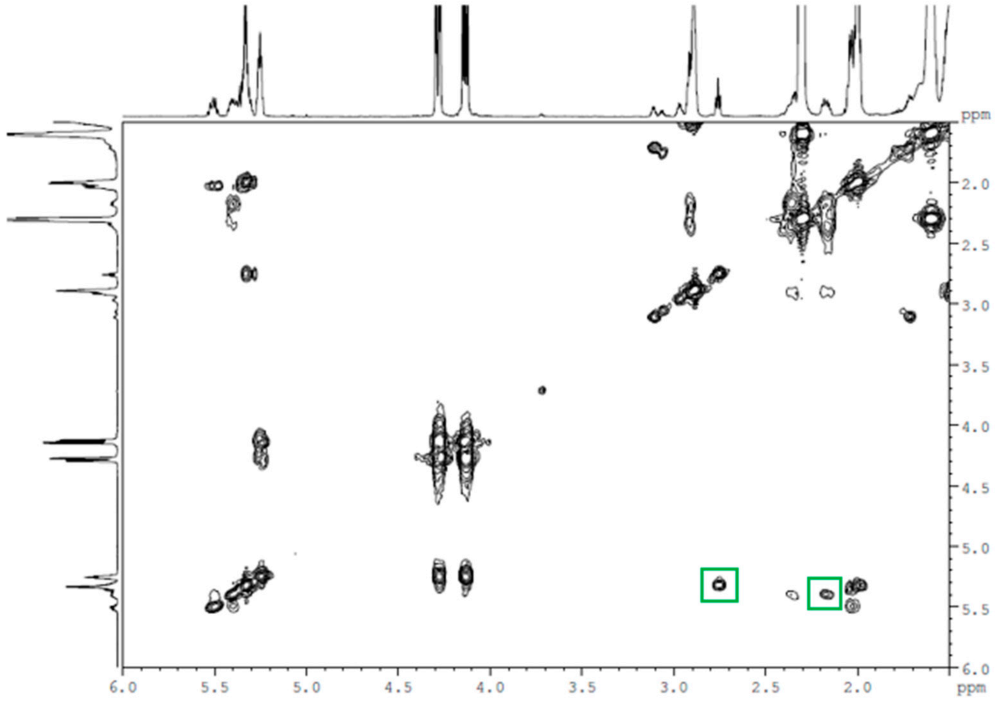


**Figure S2.** The 2D COSY spectrum of $^{55}A$

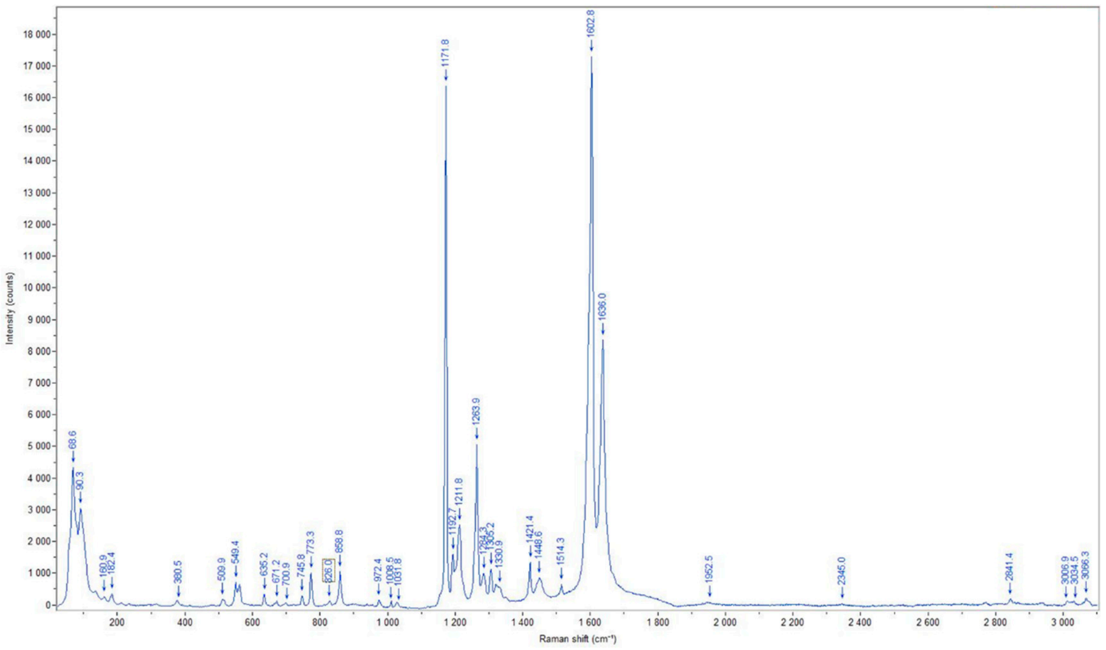


**Figure S3**. The RAMAN spectrum of *p*-MCA

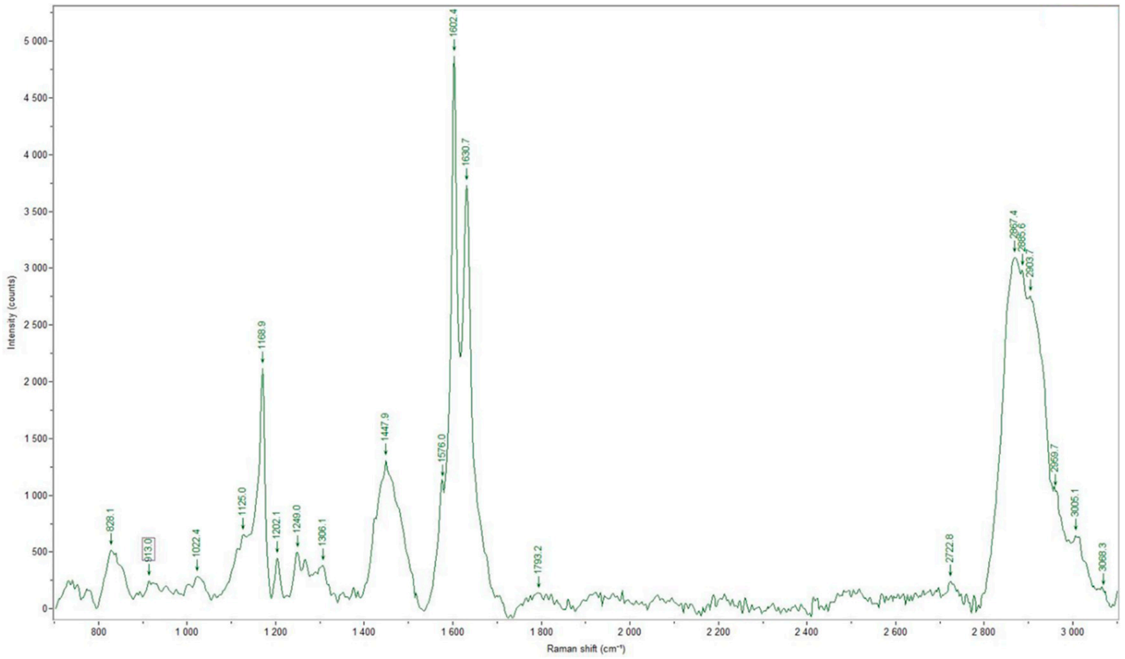


**Figure S4**. The RAMAN spectrum of $^{100}$B

**Table S1.** The SPF values for $^{55}B_y$, $^{80}B_y$ and $^{100}B_y$ at different concentrations of *p*-MCA in oleogel

| | % *p*-MCA in oleogel | Sample 1 | Sample 2 | Sample 3 | SPF Interval |
|---|---|---|---|---|---|
| $^{55}B_y$ | 10 | 7.2 | 6.6 | 7 | 6.9±0.30 |
| | 8 | 6.2 | 6.4 | 5.9 | 6.2±0.25 |
| | 6 | 4.6 | 4.1 | 4 | 4.3±0.3 |
| | 4 | 3.2 | 3.5 | 2.9 | 3.2±0.3 |
| $^{80}B_y$ | 10 | 6.7 | 6.8 | 7 | 6.8±0.15 |
| | 8 | 4.8 | 4.5 | 4.9 | 4.7±0.2 |
| | 6 | 3.5 | 3.2 | 3.5 | 3.4±0.15 |
| | 4 | 2.9 | 2.4 | 3.1 | 2.8±0.35 |
| $^{100}B_y$ | 10 | 7.0 | 7.5 | 7.1 | 7.2±0.30 |
| | 8 | 4.6 | 4.8 | 4.4 | 4.6±0.20 |
| | 6 | 4.2 | 4.9 | 4.3 | 4.45±0.35 |
| | 4 | 2.6 | 2.2 | 2.7 | 2.5±0.25 |
